# Role of the disulfide bond in stabilizing and folding of the fimbrial protein DraE from uropathogenic *Escherichia coli*

**DOI:** 10.1074/jbc.M117.785477

**Published:** 2017-07-24

**Authors:** Justyna Pilipczuk, Beata Zalewska-Piątek, Piotr Bruździak, Jacek Czub, Miłosz Wieczór, Marcin Olszewski, Marta Wanarska, Bogdan Nowicki, Danuta Augustin-Nowacka, Rafał Piątek

**Affiliations:** From the Departments of ‡ Molecular Biotechnology and Microbiology and; § Physical Chemistry, Gdańsk University of Technology, 80-233 Gdańsk, Poland,; the ¶ Nowicki Institute for Women's Health Research, Brentwood, Tennessee 37027, and; the ‖ Faculty of Chemistry, University of Gdańsk, 80-308 Gdańsk, Poland

**Keywords:** bacterial adhesion, disulfide, immunoglobulin fold, immunoglobulin-like domain, protein folding, protein stability, Dr fimbriae, DraE adhesin, chaperone-usher pathway

## Abstract

Dr fimbriae are homopolymeric adhesive organelles of uropathogenic *Escherichia coli* composed of DraE subunits, responsible for the attachment to host cells. These structures are characterized by enormously high stability resulting from the structural properties of an Ig-like fold of DraE. One feature of DraE and other fimbrial subunits that makes them peculiar among Ig-like domain-containing proteins is a conserved disulfide bond that joins their A and B strands. Here, we investigated how this disulfide bond affects the stability and folding/unfolding pathway of DraE. We found that the disulfide bond stabilizes self-complemented DraE (DraE-sc) by ∼50 kJ mol^−1^ in an exclusively thermodynamic manner, *i.e.* by lowering the free energy of the native state and with almost no effect on the free energy of the transition state. This finding was confirmed by experimentally determined folding and unfolding rate constants of DraE-sc and a disulfide bond-lacking DraE-sc variant. Although the folding of both proteins exhibited similar kinetics, the unfolding rate constant changed upon deletion of the disulfide bond by 10 orders of magnitude, from ∼10^−17^ s^−1^ to 10^−7^ s^−1^. Molecular simulations revealed that unfolding of the disulfide bond-lacking variant is initiated by strands A or G and that disulfide bond-mediated joining of strand A to the core strand B cooperatively stabilizes the whole protein. We also show that the disulfide bond in DraE is recognized by the DraB chaperone, indicating a mechanism that precludes the incorporation of less stable, non-oxidized DraE forms into the fimbriae.

## Introduction

**Figure 1.**
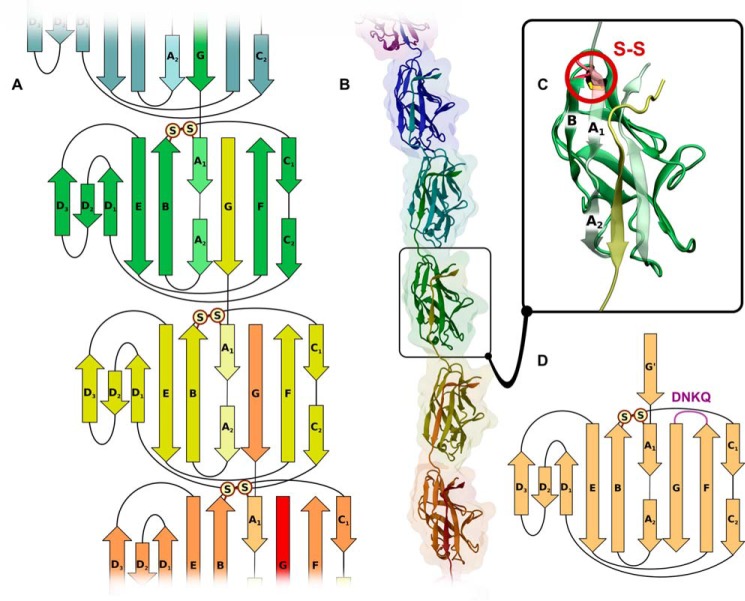
**Structural and schematic view of the DraE protein.** *A* and *B*, topological diagram and structural view of a section of the adhesive polymer shown to illustrate the DSC. *C*, a structural view of a single subunit in the context of the adhesive polymer. The location of the disulfide bond is shown explicitly (based on Protein Data Bank entry 1RXL). *D*, topological diagram of a single DraE-sc subunit with the DNKQ linker marked in *purple*. Strands G and G′ have identical sequences.

Dr fimbriae are adhesive structures responsible for the attachment of uropathogenic *Escherichia coli* to human host cells (1). Encoded by the *dra* operon and formed *in vivo* via a conserved chaperone-usher pathway of Gram-negative bacteria (2), Dr fimbriae are homopolymers of DraE adhesin proteins capped by a single DraD subunit (3). All protein subunits of the chaperone-usher adhesive organelles share a common incomplete immunoglobulin (Ig-like) fold lacking the C-terminal strand G found commonly in the classical Ig-fold (4, 5). Therefore, the DraE monomer adopts the structure of a six-stranded β-sandwich formed by the A to F β-strands with an exposed hydrophobic cleft (Fig. 1) (6, 7). Folding of DraE in the periplasm is catalyzed by DraB, a specific chaperone that forms soluble complexes with adhesin subunits (8). According to the donor strand complementation mechanism (DSC),^2^ the chaperone fills the hydrophobic cleft of DraE by inserting its donor strand G1 and forms a super-barrel structure with an extensive chaperone-subunit interface. The subsequent formation of surface-located Dr fimbriae occurs through an outer membrane assembly platform protein (usher) DraC. This process critically depends on the 16-residue-long N-terminal extension (Nte) protruding freely from the β-sandwich structure of DraE (6). During the process of Dr fimbriae extension, DraB catalyzes the donor strand exchange (DSE) reaction in which each DraE subunit donates its Nte donor strand to the preceding subunit and accepts the Nte peptide of the next subunit (Fig. 1, *A* and *B*). The DraD subunit lacks the Nte donor strand crucial for the DSE reaction and hence only exists at the tip of the fimbrial structure (3). Notably, because of the complex folding mechanism of native fimbriae, *in vitro* investigation of DraE usually relies on a recombinant self-complemented variant (DraE-sc), the minimal counterpart of a fimbrial subunit in a functional adhesive structure in which the Nte donor strand is fused at the C terminus with a short peptide linker (9, 10). In consequence, the self-complemented fimbrial subunits exist as monomeric single-domain proteins with a full Ig-like fold, in the context of which the self-complementing Nte strand is canonically referred to as the G strand. The DraE-sc adhesin used in this work is a 16.32-kDa protein in which the Nte donor strand is fused at the C terminus with a DNKQ linker (Fig. 1*D* and supplemental Fig. S1).

Because proper assembly of adhesive organelles requires the activity of the periplasmic chaperone and the outer membrane usher protein, the disjoining of two consecutive fimbrial subunits at the cell surface is irreversible and results in loss of adhesive organelles. This critical role of the adhesive organelles in the chaperone-usher system forced them to evolve to become the most stable protein structures ever described (9, 11–14). Indeed, the exceptional stability of adhesive structures has been well documented for type 1 pili, P pili, Dr/Afa-III fimbriae of *E. coli*, and F1 antigen of *Yersinia pestis* (9, 11–14). The stability of the constituent protein subunits is typically characterized by melting temperature above 80 °C, free energy of unfolding up to 85 kJ mol^−1^, and unfolding rate constant on the order of 10^−17^ s^−1^ (9, 11, 12) and stems from their peculiar structural properties, in particular a single disulfide bond joining adjacent A and B β-strands (Fig. 1). Disruption of the disulfide bond in DraE-sc by Cys → Ala mutations abolishes the high stability of the protein, lowering its melting temperature from 87 to 65 °C. Such a localization of the disulfide bond is unique for structural subunits of chaperone-usher type, as in other proteins with the Ig-fold the disulfide bond typically connects strands B and F (10). The A–B type disulfide bond is well conserved in the family of fimbrial subunits: it was found in 90% of 16,900 identified non-redundant fimbrial protein sequences (supplemental Fig. S2 and Table S1).

To date, the formation of DraE-like disulfide bond was thoroughly investigated in FimC chaperone-catalyzed folding of FimA major subunit of type 1 pili (15). In *in vitro* experiments, the FimC chaperone could catalyze the folding of FimA with an intact disulfide bond, but not in its reduced form. This means that FimC only recognizes and processes FimA subunits that were earlier oxidized by the periplasmic oxidoreductase DsbA (15). This mechanism ensures that only the ultrastable protein subunits are incorporated into the emerging pili. Despite the prevalence of disulfide bonds in the structures of bacterial adhesins, the mechanism of chaperone-mediated quality control of disulfide bond formation was directly verified experimentally only for FimC, a member of the short F1-G1 loop (FGS) chaperone subfamily, which includes chaperones aiding in the assembly of well structured, heteropolymeric, and monoadhesive pili (16, 17). DraB, on the contrary, belongs to the long F1-G1 loop (FGL) subfamily, which includes chaperones participating in biogenesis of generally amorphic, homopolymeric, and polyadhesive structures. Interestingly, Caf1M from *Y. pestis*, one of the most widely studied FGL-type chaperones, catalyzes the formation of F1 adhesive capsular antigen composed of Caf1 protein subunits that do not possess any disulfide bond in their structure (18). This means that despite the high structural and functional conservation of chaperone proteins, the disulfide bond quality control mechanism is not conserved in the whole family and requires further investigation.

In this paper, we investigate the mechanism by which the disulfide bond affects the stability and folding of DraE protein. We focus on the following questions: 1) How does the disulfide bond participate in generation of the enormously high stability of DraE protein: by modulation of the free energy of the transition state (kinetic stabilization) or by stabilization of the native conformation with respect to the unfolded state (thermodynamic stabilization)? 2) Does the presence of the disulfide bond significantly alter the folding pathway of DraE protein *in vitro*, similarly to the drastically affected unfolding pathway? 3) Is the disulfide bond control mechanism, previously described in the folding of FimA by the FGS-type FimC chaperone, also at play in the folding of DraE by the FGL-type DraB chaperone? The answers to these questions provide a unique insight into how the A–B type disulfide bond affects the folding and stability of the DraE, the member of a conserved family of chaperone-usher type fimbrial subunits that allows for stable bacterial adhesion under shear stress in urine flow, ultimately paving way for pathogenesis. Our conclusions can be generalized to shed light on, as well as possibly modulate or engineer, the folding and stability of proteins with the common Ig-like fold.

## Results

### A–B type disulfide bond has little effect on the kinetics of DraE-sc folding

**Figure 2.**
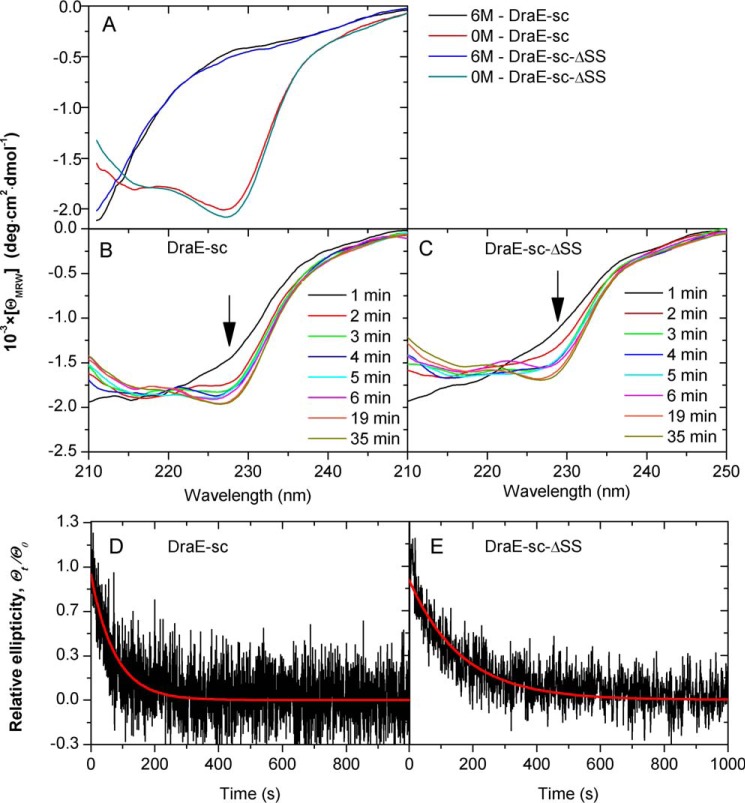
**Kinetics of DraE-sc and DraE-sc-ΔSS refolding.** *A*, far-UV CD spectra of both protein variants in their native (0 m) and denatured (6 m GdmCl) state. *B* and *C*, protein refolding monitored at the respective time by the shape of far-UV CD spectra, initiated by 100-fold dilution of initial solutions of proteins in 6 m GdmCl. *D* and *E*, changes in relative ellipticity measured at 227 nm during the refolding experiments. *Red lines* denote the fitted first rate kinetic exponential function.

To investigate the effect of the A–B type disulfide bond on the folding of DraE, we compared the refolding kinetics of DraE-sc (with intact disulfide bond) and DraE-sc-ΔSS (with Cys → Ala mutations) denatured in 6 m guanidinium hydrochloride (GdmCl) using far-UV CD spectroscopy (protein sequences of all DraE variants used in this work are presented in supplemental Fig. S1). The spectra of native DraE-sc and DraE-sc-ΔSS in sodium phosphate buffer (pH 7.5) are very similar and exhibit two minima at 228 and 215 nm, confirming the marginal effect of Cys → Ala mutations on the tertiary structure of DraE-sc (Fig. 2*A*). At the same time, the virtually featureless spectra of both proteins in buffer containing 6 m GdmCl confirm their proper unfolding (Fig. 2*A*). The refolding of both protein variants, initiated by 100-fold manual dilution of samples with sodium phosphate buffer, was analyzed by recording far-UV CD spectra in the 203–250-nm range (Fig. 2, *B* and *C*) and by monitoring the CD signal at a single wavelength of 227 nm (Fig. 2, *D* and *E*). The spectra of DraE-sc and DraE-sc-ΔSS obtained 1 min after the initiation of refolding are characteristic of the unfolded state, with negative slope and no local minima (Fig. 2, *B* and *C*). Between 2 and 6 min after initiation, the spectra begin to exhibit curvature typical for native-like proteins, and within 20 min after initiation of refolding, they are identical to the spectra of fully folded DraE-sc (Fig. 2, *A–C*). These time-resolved measurements clearly suggest that the refolding of DraE-sc and DraE-sc-ΔSS is rather slow and follows very similar kinetics.

The actual refolding rates of both proteins were determined by monitoring the far-UV CD signal at 227 nm (Fig. 2, *D* and *E*). The signals for both DraE-sc and DraE-sc-ΔSS fitted well to a single exponential first-order kinetics function, yielding rate constants *k*_fold_ of 0.013 ± 0.001 and 0.006 ± 0.001 s^−1^ that correspond to folding half-times of 52 and 113 s, respectively. This confirms that the presence of the A–B type disulfide bond has little effect on the DraE-sc folding rate, in contrast to its high impact on the unfolding rate (10).

### A–B type disulfide bond has marginal effect on the transition state free energy in DraE-sc folding

The folding rate is strictly related to the height of free energy barrier, Δ*G*_U→‡_, defined as the difference between the free energy of transition (‡) and unfolded (*U*) states, according to the Equation 1,

(Eq. 1) $$k_{\text{fold}}=\kappa \frac{k_{\text{B}}T}{h}e^{\frac{-\Delta G_{\text{U}\to ‡}}{RT}}$$

where κ is the transmission coefficient, *k*_B_ is the Boltzmann constant, *h* is the Planck's constant, and *R* is the gas constant.

The 2-fold faster folding of DraE-sc compared with DraE-sc-ΔSS indicates that the folding free energy barrier, Δ*G*_U→‡_, is lower for the protein with disulfide bond. The change in the height of the free energy barrier associated with deletion of the disulfide bond, ΔΔ*G*_U→‡_, is given by the Equation 2,

(Eq. 2) $$△△G_{\text{U}\to ‡}=△G_{\text{U}\to ‡}-△G_{\text{U}\to ‡}^{*}=RT\;\text{ln}\frac{K_{\text{fold}}^{*}}{K_{\text{fold}}}$$

where *G*_U→‡_ and *G*_U→‡_^*^ (*k*_fold_ and *k*_fold_^*^) are the free energy barriers (rate constants) of folding for DraE-sc and DraE-sc-ΔSS, respectively.

The calculated ΔΔ*G*_U→‡_ is equal to −1.91 ± 0.42 kJ mol^−1^ at 25 °C. If the free energies of the unfolded states were identical, this value would be exactly equal to the difference between the transition state free energies of wild-type and mutant DraE-sc. This is, however, not the case, because the presence of a loop between two cysteine residues in the wild-type protein lowers the entropy of the unfolded state according to the Equation 3,

(Eq. 3) $$\Delta S=-2.1-\frac{3}{2}ln\left(n\right)$$

where Δ*S* is the decrease of configurational entropy associated with the presence of a loop, and *n* is the number of loop residues (19). In DraE-sc, there are 33 residues between both cysteine residues, so that the −ΤΔ*S* contribution to the free energy of the unfolded state of DraE-sc with respect to DraE-sc-ΔSS (*G*_U_ − *G*_U_^*^) at 25 °C is equal to +2.2 kJ mol^−1^. Hence, the value of ΔΔ*G*_U→‡_ determined above (−1.91 ± 0.42 kJ mol^−1^) can be almost solely attributed to the difference in unfolded states of proteins, showing that the presence of the A–B type disulfide bond has virtually no effect on the free energy of the transition state. This is also supported by the Φ-value analysis that allows evaluation of the importance of a particular residue in stabilizing the transition state structure (supplemental materials).

The DraE-sc protein is characterized by an extraordinarily high kinetic stability expressed in the activation energy of unfolding of 460 kJ mol^−1^ and, consequently, an exceptionally low unfolding rate constant of 10^−17^ s^−1^ at 25 °C (9). This kinetic stability is directly related to the height of free energy barrier of unfolding, Δ*G*_N→_**_‡_**, here defined as the free energy difference between the transition (‡) and native (*N*) states. Accordingly, the disulfide bond may enhance the kinetic stability of DraE-sc in two ways, either by increasing the free energy of the transition state or decreasing the free energy of the native state. Notably, both mechanisms elevate the activation free energy barrier of unfolding, Δ*G*_N→_**_‡_**, but only the former leads to a significantly slower folding rate. As shown above, the disruption of the A–B type disulfide bond has no effect on the free energy of the transition state, ruling out the mechanism in which the disulfide bond enhances kinetic stability of DraE by elevating the free energy of the transition state.

### Cystine reduction abolishes the exceptional stability of DraE-sc

Consequently, the folding kinetics of DraE-sc and DraE-sc-ΔSS strongly suggests that the stabilizing effect of the A–B type disulfide bond results from the stabilization of the native state with respect to the unfolded state, *i.e.* negative ΔΔ*G*_N→U_. To verify this hypothesis, we first analyzed whether the high stability characteristic of the native disulfide bond–containing DraE-sc is exclusively dependent on the oxidation state of the cysteine/cystine residues.

**Figure 3.**
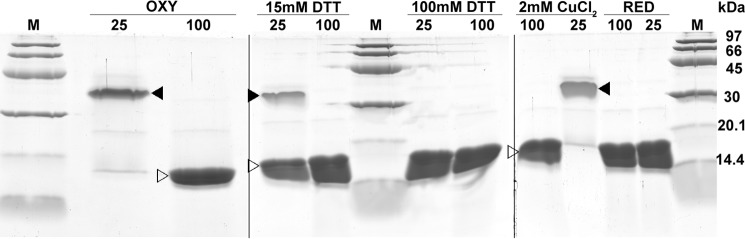
**Influence of reoxidation of DraE-sc on its structure-dependent retardation in SDS-PAGE.** *Lanes 100* and *25*, samples incubated with Laemmli buffer at 100 and 25 °C, respectively, followed by electrophoresis (SDS–15% polyacrylamide gel). The *filled arrowhead* denotes retarded form of DraE-sc protein that remains folded during electrophoresis and in consequence binds fewer SDS molecules than the denatured protein, denoted by an *open arrowhead*. At 25 °C samples of stable oxidized (disulfide bond–containing) DraE-sc protein are retarded in contrast to it reduced (disulfide-lacking) form. At 100 °C samples of both oxidized and reduced forms of DraE-sc are fully denatured. *OXY*, sample of native non-denatured (disulfide bond–containing) DraE-sc protein. This sample was used as a substrate to obtain denatured and reduced DraE-sc in 6 m GdmCl and 50 mm DTT. *RED*, sample of refolded reduced (disulfide bond lacking) DraE-sc protein. This sample was further subjected to air oxidation under different conditions. *15 mm DTT*, sample of refolded reduced DraE-sc protein after 7 days of air oxidation in 15 mm DTT. *2 mm CuCl_2_*, sample of refolded reduced DraE-sc protein after 7 days of air oxidation in 2 mm CuCl_2_. *100 mm DTT*, sample of refolded reduced DraE-sc protein after 7 days of air oxidation in 100 mm DTT. *M*, low-molecular-mass calibration kit for SDS electrophoresis (97, 66, 45, 30, 20.1, and 14.4 kDa; GE Healthcare); note that because of differences in conformational states, protein masses need not correspond to marker positions.

**Figure 4.**
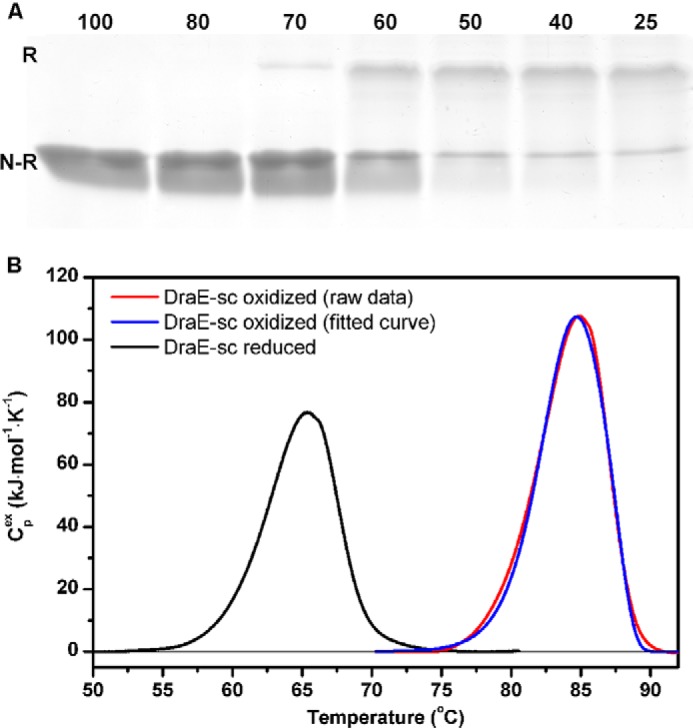
**Stability of refolded reoxidized (disulfide bond–containing) DraE-sc.** *A*, examination of kinetic stability of the reoxidized DraE-sc protein using an SDS resistance assay. Protein samples were incubated in Laemmli buffer at the indicated temperatures (see above gel lanes) for 10 min immediately prior to loading onto the gel. *N-R*, non-retarded unfolded form of the protein; *R*, native-like retarded form of the protein. The SDS-PAGE retardation assay of refolded reduced (–SH group containing) DraE-sc is included in Fig. 3 (*RED*). *B*, investigation of thermal stability and mechanism of thermal unfolding of reoxidized DraE-sc protein by differential scanning calorimetry. Shown is the experimental dependence of excess heat capacity (*C*_p_^ex^) on temperature for the reoxidized DraE-sc protein, obtained at a scanning rate of 1.0 °C min^−1^, and the experimental curve fitted after baseline subtraction to the two-state kinetic model of protein unfolding. For comparison, the denaturation curve of refolded reduced DraE-sc protein was included in the thermogram.

To this end, the DraE-sc protein was denatured in reducing environment by incubation in Tris-HCl (pH 8.0) buffer containing 6 m GdmCl and 50 mm DTT, and subsequent refolding of DraE-sc was initiated by 100-fold dilution that yielded final DTT concentration of 15 mm. Analysis of the sample with analytical size-exclusion chromatography confirmed the elution of reduced (containing the –SH group) refolded DraE-sc protein with retention time identical to that for the non-denatured protein. The stability of refolded reduced DraE-sc was checked using the SDS-PAGE retardation test in which protein samples mixed with Laemmli buffer were incubated at 25 °C or heated at 100 °C for 10 mins before electrophoresis. This technique is based on the retardation of proteins that maintain tertiary structure during electrophoresis and in consequence bind fewer SDS molecules than the same proteins fully denatured by boiling in Laemmli buffer (8, 10). The refolded reduced DraE-sc incubated in Laemmli buffer at 25 °C did not exhibit any retardation in the SDS-PAGE and migrated identically as in the heated sample, indicating susceptibility to SDS-induced denaturation (Fig. 3, *RED*). In contrast, the non-denatured DraE-sc protein with intact disufide bond (treated as a control) incubated in Laemmli buffer at room temperature was significantly retarded with respect to the heated sample because it was apparently not denatured by SDS at 25 °C (Fig. 3, *OXY*). The obtained refolded reduced DraE-sc protein denatured thermally at 65 °C in the differential scanning calorimetry experiment, identically as the DraE-sc-ΔSS mutant (Fig. 4*B*) (10). These experiments confirm that the reduced DraE-sc loses its high stability characteristic for disulfide-containing DraE-sc and that its stability is identical to that previously reported for DraE-sc-ΔSS (10).

### Reoxidation restores high stability of DraE-sc

Next we checked whether reoxidation of the cysteine pair to cystine restores the stability of reduced refolded DraE-sc to that of the protein in its native state. The air oxidation was performed in two modes at room temperature for 7 days, using a sample of reduced DraE-sc in refolding buffer containing 15 mm DTT. In the first mode, the refolded reduced DraE-sc was slowly oxidized in air. In the second mode, air oxidation of refolded reduced DraE-sc was catalyzed by 2 mm CuCl_2_. In the control experiment, air oxidation was prevented by the presence of 100 mm DTT. All three samples were monitored every 24 h for the presence of oxidized DraE-sc using the SDS-PAGE retardation test as described above. In control samples (100 mm DTT) collected on the seventh day, incubation in Laemmli buffer at 25 and 100 °C yielded identical, non-retarded bands on the gel characteristic for the reduced form, confirming that high concentration of DTT blocked air oxidation of refolded reduced DraE-sc (Fig. 3, *100 mm DTT*). In contrast, in the sample taken 1 day after initiation of the CuCl_2_ experiment, incubated before electrophoresis in Laemmli buffer at 25 °C, a retarded band was observed that migrated identically as the stable oxidized DraE-sc. In quantitative terms, 100% of soluble DraE-sc was found to be oxidized after 24 h of incubation (Fig. 3, *2 mm CuCl_2_*). Samples from the non-catalyzed air-oxidation experiment taken after 7 days contained 30% of oxidized DraE-sc, as indicated by the intensity of the retarded and non-retarded bands (Fig. 3, *15 mm DTT*). We therefore conclude that the formation of disulfide bond in the structure of fully folded reduced DraE-sc is sufficient to restore to it stability.

The refolded and reoxidized disulfide-containing DraE-sc protein was further analyzed to determine its stability relative to DraE-sc that was not denatured and reduced. First, we performed a variation of the SDS-PAGE retardation test with different incubation temperatures in Laemmli buffer: 25, 40, 50, 60, 70, 80 and 100 °C. The retarded SDS-resistant DraE-sc protein was observed in samples incubated at up to 60–70 °C (Fig. 4*A*, compare with Fig. 3, *RED* for reduced refolded DraE-sc), meaning that the oxidized form was largely resistant to the combination of elevated temperature and denaturing conditions. Indeed, thermal analysis of the reoxidized sample of DraE-sc by differential scanning calorimetry confirmed that the formation of disulfide bond restored the high stability of the protein. The refolded reoxidized DraE-sc heated with a scan rate of 1.0 °C min^−1^ unfolded at *T*_m_ = 85.1 °C. The denaturation was irreversible, with scan rate–dependent kinetics typical for proteins whose unfolding is kinetically controlled. The obtained calorimetric data were then analyzed using the kinetic two-state reduced form of the Lumry-Eyring model, yielding activation energy for unfolding equal to *E*_a_ = 465.4 ± 17.3 kJ mol^−1^ and rate constant *k*_obs_ = 1.80 10^−16^ s^−1^ that corresponds to the unfolding half-time τ_½_ of 10^8^ years at 25 °C (Fig. 4*B*). These data are almost identical to those reported previously for DraE-sc with an intact disulfide bond (9). Presented reoxidation experiments clearly confirm that the mechanism of DraE-sc stabilization by disulfide bond is strictly connected with the effect exerted on its final folded structure.

### MD simulations reveal the unfolding pathways of DraE

**Figure 5.**
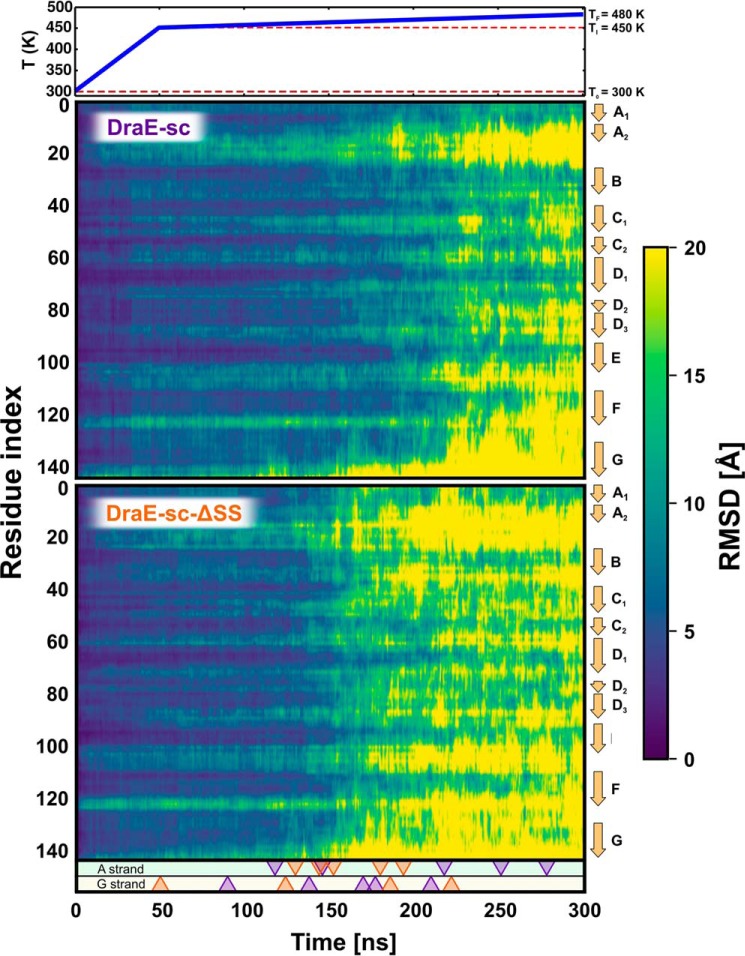
**Per-residue RMSD of atomic positions in thermal denaturation MD simulations of DraE-sc and DraE-sc-ΔSS.** The *top panel* shows the temperature profile used to enforce thermal unfolding over the course of 300 ns. RMSD values, representing deviation from the native structure, were averaged over five independent simulation runs. *Triangles* indicate strand unfolding events in which strand A (*top panel*) or strand G (*bottom panel*) lose their secondary structure. These triangles are color-coded according to the presence or absence of the disulfide bond; *e.g.* the position of an orange top triangle corresponds to the simulation time at which strand A dissociated in the reduced protein form (without the disulfide bond).

To gain an atomistic insight into the process of thermal unfolding, we ran a series of 10 independent MD simulations in which two forms of the DraE-sc protein, reduced (lacking the disulfide bond) and oxidized (containing the disulfide bond), were subject to thermal unfolding. Given the short time scales accessible to MD simulations, the *in silico* thermal unfolding was carried out in temperatures higher than the experimentally determined *T*_m_, with a short initial period of heating from 300 to 450 K and subsequent slow annealing from 450 to 480 K (Fig. 5). To monitor the deviation from the native structure, we traced the root mean square deviation (RMSD) of the protein residues with respect to the folded state (Fig. 5).

By inspection of the residue-wise RMSD values, averaged over all simulations, one can see that in the reduced form (*bottom panel*), the C-terminal strand G and the flexible region connecting strands A and B are the first to lose their native structure, so that the denaturation of the entire protein domain is initiated by an increase in the conformational flexibility of these two regions. The subsequent unfolding of the core region (strands B–F) occurs cooperatively but is delayed by 20–50 ns, consistently with the previously described unfolding pathway of Ig-like domains (20). On the other hand, in presence of the disulfide bond, the appearance of large deviation from the native structure (mean RMSD > 15 Å) is offset globally by additional 50 ns. This means that the disulfide bond not only stabilizes the A/B joining segment, which now dissociates on average 50 ns later than in the reduced form, but also cooperatively enhances the stability of the whole protein by significantly delaying the onset of denaturation (see supplemental Movies S1 and S2 for illustration of sample unfolding pathways).

This cooperative effect can be explained by noting that particularly in the case of kinetically controlled unfolding, denaturation has to start with a single thermal excitation that exposes the hydrophobic core of the protein (21), so that the thermal stability can be enhanced by reducing the extent of structural fluctuations in the folded state. However, we observed that even almost complete displacement of strand G did not necessarily lead to denaturation of the oxidized (disulfide bond–containing) protein. The strand could still return to its initial position in the hydrophobic cleft, suggesting that the disulfide bond not only limits the extent of thermal fluctuations but also reduces the protein susceptibility to denaturation when fluctuations occur anyway, hence enhancing kinetic stability.

### Mechanical unfolding simulations explain the placement of the disulfide bond

**Figure 6.**
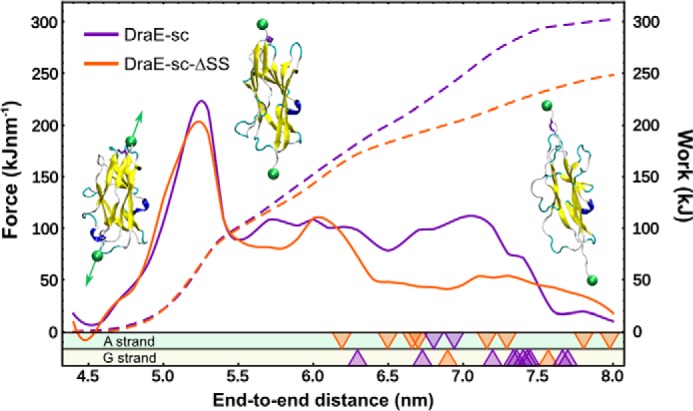
**Force-extension curves from mechanical unfolding MD simulations (*dashed lines*), shown along with the unfolding work profiles (*solid lines*; equal to the integral of the pulling force) of DraE-sc and DraE-sc-ΔSS.** Sample structures corresponding to the respective end-to-end distances are shown, with *green spheres* indicating the centers of mass used to define this distance coordinate. Note that the chosen pulling direction, marked with *arrows*, should correspond to forces exerted on an extended fimbrial polymer when bacteria are subject to shear forces. All values are averaged over 10 independent simulation runs. *Triangles* indicate strand dissociation events in which strand A (*top*) or strand G (*bottom*) detach from the core B–F region in individual simulations and are color-coded according to the presence or absence of the disulfide bond; *e.g.* the position of an *orange top triangle* corresponds to an end-to-end distance at which strand A dissociated in the reduced protein form (without the disulfide bond).

It should be noted that in the actual biological system, *i.e.* in the context of an adhesive DraE homopolymer, it is the G strand that, according to the DSE reaction mechanism, critically determines the stability of the entire fimbrium. Although the thermal unfolding simulations clearly suggest that the stabilization of strand A delays the global loss of a definite fold, it is not obvious that it also enhances the fimbrium's resistance to mechanical stress. Hence, to address this issue, we performed a set of 20 independent steered MD simulations in which the C and N termini of both DraE variants were pulled apart using external force, as would happen if the fimbrium was stretched because of shear forces acting on the adherent bacterial cell. The results, along with a schematic system setup, are shown in Fig. 6, in which the *dashed line* denotes the pulling force averaged over 10 simulations, and the *solid line* represents the work done by the pulling force (the integral of the mean force). We also monitored strand dissociation events, shown at the *bottom* of the plot as *purple* and *orange arrowheads* that denote the end-to-end distance at which A or G strand dissociated from the protein, *i.e.* lost more than 50% of the contacts formed in the native state.

As seen in Fig. 6, the protein domain responds elastically to the applied force as the displacement increases to 5.2 nm. At this end-to-end distance, after the force peaks at a maximum of ∼200 kJ mol^−1^ nm^−1^ (equivalent to ∼330 pN), strands A and G become shifted with respect to each other and expose a hydrophobic cleft. The disulfide-induced stabilization does not become apparent until this point, and indeed mean work required to stretch the two forms starts to differ only above 5.5 nm, eventually (>7.0 nm) diverging by more than 20%. Further pulling leads to steady loss of secondary structure with no additional characteristic events, until one of the pulled strands detaches from the Ig-like fold, eventually causing the pulling force to decrease and, consequently, the work to level off. Importantly, in the absence of the disulfide bond, the A strand dissociates more easily than G, as evidenced by the distribution of *arrowheads* at the bottom of Fig. 6, where 8 of 10 strand displacement events (*orange*) correspond to strand A. This pattern changes when the disulfide bond is present, with G strand dissociating in 10 of 12 cases (purple) and majority of events occurring above 7.0 nm.

One can thus note that the main conclusion of the thermal denaturation simulations—that stabilization of strand A delays the unfolding of the entire protein—translates to the mechanical unfolding. Even more importantly, the results show that, in the absence of the disulfide bond, strand A is the first to dissociate upon application of mechanical force, so that, given the cooperative character of the unfolding process, the placement of the A–B disulfide bond appears to be an adaptation to function under shear stress.

### Only DraE subunits with an intact disulfide bond are incorporated into Dr fimbriae

**Figure 7.**
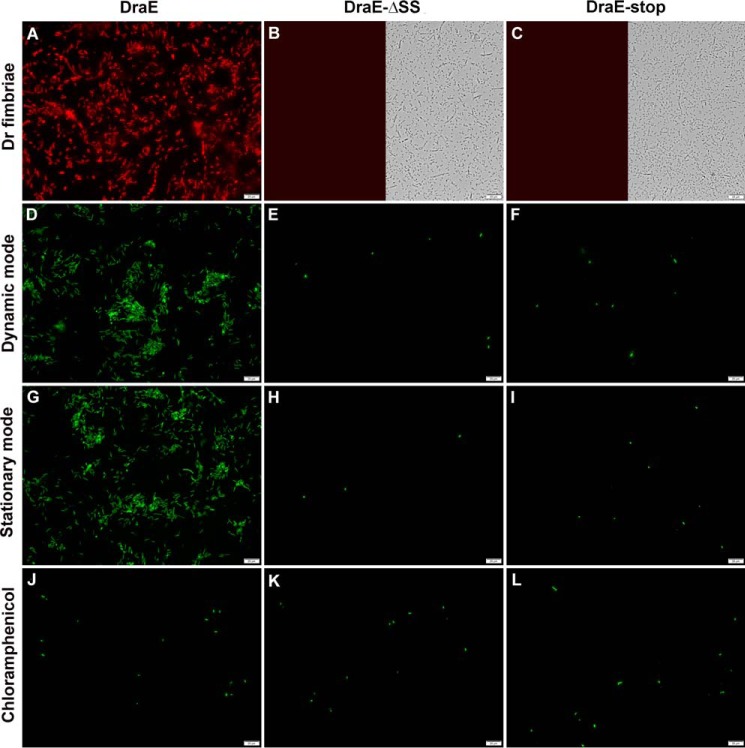
**Detection of surface located Dr fimbriae in the *E. coli* AAEC191 strains encoded in the pCC90, pCC90DraE-ΔSS, and pCC90D54stop (DraE negative mutant) plasmids, denoted as *DraE*, *DraE*-Δ*SS*, and *DraE-stop*, respectively.** *A–C*, for Dr fimbriae, immunofluorescence examination with rabbit anti-DraE and goat TRITC-labeled anti-rabbit antibodies of Dr fimbriae at the surface of AAEC191A/pCC90 (*A*), AAEC191A/pCC90DraE-ΔSS (*B*), and control AAEC191A/pCC90D54stop (*C*) strains. The *left* and *right halves* of *B* and *C* represent fluorescence and white light views of the same bacterial sample. *D–L*, microscopic fluorescence detection of Dr fimbriae-mediated adherence of AAEC191A/pCC90/pGFP (*D*, *G*, and *J*), AAEC191A/pCC90DraE-ΔSS/pGFP (*E*, *H*, and *K*) and control AAEC191A/pCC90D54stop/pGFP (*F*, *I*, and *L*) strains to cancer T24 urinary bladder cells. Bacterial adherence were performed in three experiments. In stationary mode (*G–I*), the cell line was incubated for 20 min with bacterial suspension without any shaking. In dynamic mode (*D–F*), bacterial suspension was passed over the cell line in enforced flow conditions for 20 min to generate shear stress of 0.1 pN μm^−2^. With chloramphenicol (*J–L*), adherence performed identically as in *stationary mode*, but the bacterial cells before addition to T24 cells were preincubated in medium containing 300 μm of chloramphenicol to block receptor binding sites on the Dr fimbriae. Adhered bacteria were visualized by GFP-dependent green fluorescence. The *bars* denote 20 μm. Images are representative of three independent experiments.

Because the A–B type disulfide bond critically determines the stability of DraE, as shown above, we checked whether DraE-ΔSS (non-self-complemented, supplemental Fig. S1) will be a proper substrate for the fimbrial assembly machinery. Using the *E. coli* AAEC191A/pCC90 strain that encodes the whole *dra* operon on the pCC90 plasmid and constitutively produces Dr fimbriae (22), we constructed a modified AAEC191A/pCC90DraE-ΔSS strain that expresses wild-type DraB chaperone, DraC usher, and DraD tip fimbrial subunit along with DraE-ΔSS (all bacterial strains used in this work are summarized in supplemental Table S2). Western blotting of the periplasmic fraction from the DraE-ΔSS-expressing strain confirmed production of ∼20% less mutant adhesin relative to the strain expressing wild-type DraE (supplemental Fig. S3). The AAEC191A/pCC90D54stop (DraE negative mutant) strain was used as a control. We used immunofluorescence microscopy with polyclonal rabbit anti-DraE and goat anti-rabbit TRITC-labeled antibodies to detect surface-located Dr fimbriae in all strains. In contrast to the bacteria-producing native DraE (Fig. 7*A*), no Dr fimbriae was detected on the surface of DraE-ΔSS-expressing and control strains (Fig. 7, *B* and *C*).

To further verify that the DraE-ΔSS protein cannot form Dr fimbriae on bacterial surface, we analyzed the adhesion of fluorescent *E. coli* strains to urinary bladder cancer cells, to which Dr fimbriae specifically bind via the surface located CD55, CD66a, CD66c, and CD66e human protein receptors (23). To this end, we prepared AAEC191A/pCC90/pGFP (expressing native DraE), AAEC191A/pCC90DraE-ΔSS/pGFP (expressing DraE-ΔSS), and AAEC191A/pC90D54stop/pGFP (negative control) strains comprising the pGFP plasmid that gives constitutive expression of the GFP protein, allowing the real-time observation of bacterial adhesion with fluorescence microscopy. The adhesion experiments were performed in two modes. In the stationary assay, bacteria suspended in a cancer cell line medium with optical density (*A*_600_) of 0.1 were incubated with confluent layer of bladder cells for 20 min without any shaking. In the dynamic assay, a layer of bladder cells was exposed to identical bacterial suspensions in flow conditions for 20 min, at shear stress of 0.1 pN μm^−2^ typical for urine flow in human bladder. The strain expressing native DraE adhered efficiently to bladder cells in both experimental modes with the typical diffusion pattern. In the stationary and dynamic experiments 825 ± 69 and 923 ± 76 DraE-producing bacterial cells were attached in the area of 92,000 μm^2^ (Fig. 7, *D* and *G*). Adherence of these bacteria to host cells was specifically blocked by chloramphenicol, known to interact with receptor binding sites on the DraE subunits (7, 24): 10 min of preincubation in medium containing 300 μm chloramphenicol reduced the adherence to bladder cells to 15 ± 8 bacteria in the area of 92,000 μm^2^ (Fig. 7*J*).

Correspondingly, both the DraE-ΔSS expressing and the negative control strains exhibited almost no adherence to the bladder cells. In both adherence experiments, these strains exhibit attachment of 8 ± 5 bacteria in the area of 92,000 μm^2^ of confluent bladder cell line (Fig. 7, *E*, *F*, *H*, and *I*).

### DraE lacking disulfide bond does not oligomerize in periplasm

Earlier works showed that co-expression of DraB chaperone and disulfide bond–containing DraE in absence of DraC usher results in an accumulation of linear DraE oligomers in periplasm (8). These oligomers are products of the DSC reaction catalyzed by DraB and subsequent DSE reaction dependent on the presence of Nte peptide extension in DraE subunits. The oligomers are structurally identical to mature Dr fimbriae and exhibit high resistance to thermal and chemical denaturation. Consequently, we checked whether the DraB chaperone will catalyze formation of DraE-ΔSS oligomers in the bacterial periplasm.

**Figure 8.**
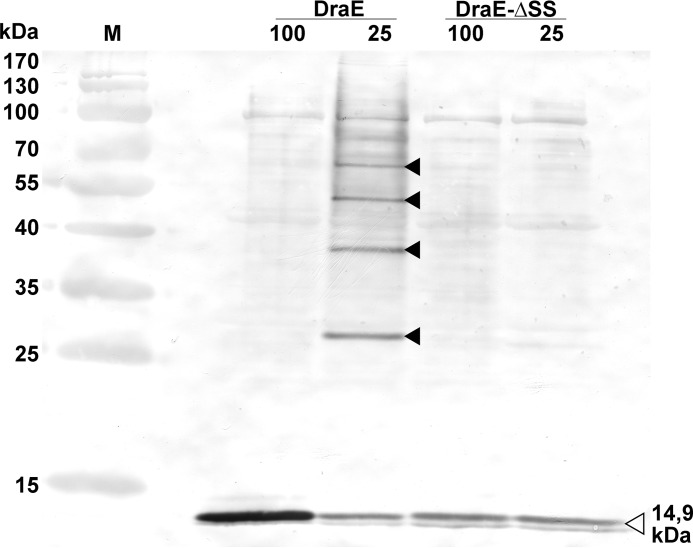
**Western blot detection of potential DraE and DraE-ΔSS oligomers in the periplasmic fractions isolated from *E. coli* BL21(DE3)/pET30b-sygDraBE and BL21(DE3)/pET30b-sygDraBE-ΔSS strains, respectively.** Samples in *lanes 100* and *25* were incubated with Laemmli buffer at 100 and 25 °C, respectively, followed by electrophoresis (SDS–15% polyacrylamide gel). The *open* and *filled arrowheads* denote monomeric fully unfolded and oligomeric SDS-resistant forms of DraE, respectively. Immunodetection was performed using primary rabbit polyclonal anti-DraE antibodies, secondary goat labeled with horseradish peroxidase anti-rabbit antibodies and diaminobenzidine as a reaction substrate. *Lane M* contained a PageRuler prestained protein ladder (Fermentas), which included 10-, 15-, 25-, 35-, 40-, 55-, 70-, 100-, 130-, and 170-kDa compounds.

We investigated this in direct *in vivo* experiments by constructing an *E. coli* BL21(DE3)/pET30b-sygDraBE-ΔSS strain that co-expressed DraB and DraE-ΔSS in periplasm. As a control, we used an *E. coli* BL21(DE3)/pET30b-sygDraBE strain encoding the wild-type DraB chaperone and the wild-type DraE fimbrial subunit with an intact disulfide bond (8). The oligomers were detected in isolated periplasmic fractions using the SDS-PAGE retardation test combined with Western blotting technique. This technique is based on the observation that unheated in Laemmli buffer periplasmic DraE oligomers are retarded in SDS-PAGE gels, because during electrophoresis they maintain their quaternary structure and thus bind fewer SDS molecules than the same proteins fully denatured by boiling in Laemmli buffer (8, 10). Analysis of periplasmic fractions isolated from the DraB/DraE-ΔSS–expressing strain using Western blotting with anti-DraE antibodies indicates that DraE-ΔSS oligomers do not form in periplasm. Both boiled and unheated samples exhibited only a single band corresponding to the (non-retarded) monomer of DraE-ΔSS subunit (Fig. 8). At the same time, DraE oligomers readily formed in periplasmic fractions isolated from the wild-type DraB/DraE-expressing control strain (Fig. 8). Western blotting analysis of the periplasmic fractions show that the DraB/DraE-ΔSS–expressing strain produces ∼30% less DraE than the control strain. We speculate that this might result from cellular instability of DraE-ΔSS, which is not folded by the DraB chaperone to its native form. Notably, the self-complemented variants DraE-sc and DraE-sc-ΔSS are produced at comparable level in the periplasm of *E. coli* BL21(DE3), ruling out the influence of Cys → Ala mutations as a possible cause of decrease in production of DraE-ΔSS (10). Based on the presented data, we conclude that DraE-ΔSS is not recognized as a proper substrate by the DraB chaperone and consequently does not form fimbrial structures on the bacterial surface.

## Discussion

**Figure 9.**
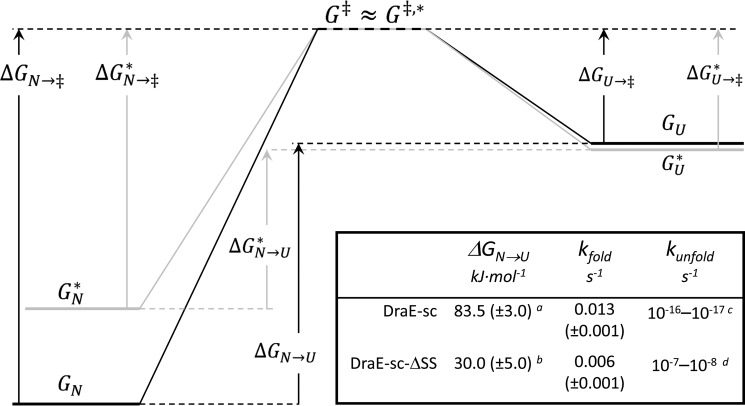
**The free energy diagram of DraE-sc (*without asterisk*, *black lines*) and DraE-sc-ΔSS (with *asterisk*, *gray lines*) folding/unfolding.** *N* corresponds to native states of proteins, *U* corresponds to unfolded states, and ‡ corresponds to the transition state. Δ*G*_N→_**_‡_** denotes the free energy barrier for unfolding, and Δ*G*_U→‡_ denotes the free energy barrier of folding. *Inset*, thermodynamic and kinetic parameters of DraE-sc and DraE-sc-ΔSS denaturation, determined or calculated for 25 °C. *^a^*, calculated on the basis of *k*_unfold_ and *k*_fold_: Δ*G*_N→U_ = −*RTln*(*k*_unfold_/*k*_fold_); *^b^*, value taken from Ref. 10; *^c^*, taken from Ref. 9; *^d^*, calculated on the basis of Δ*G*_N→U_ and *k*_fold_: *k*_unfold_ = *k*_fold_·*exp*(−Δ*G*_N→U_/*RT*).

In this work, we elaborate on the stabilizing effect of the A–B type disulfide bond, whose localization in fimbrial subunits between the A and B β-strands is unique in the context of the canonical Ig-fold (Fig. 1 and supplemental Fig. S2). As an example, we used DraE-sc, the self-complemented DraE subunit of Dr fimbriae encoded by the *dra* gene cluster of uropathogenic *E. coli*. In Fig. 9, we show a full free energy diagram of DraE-sc folding/unfolding that summarizes all available data on the effect of the A–B type disulfide bond on the stability of the DraE-sc protein. Based on prior work, we assumed a two-state mechanism of DraE-sc folding/unfolding involving native and denatured forms of the protein (9, 10). As shown in Fig. 9, the unfolding of the native disulfide bond–containing DraE-sc is very unfavorable, as indicated by the unfolding free energy of ∼83.5 ± 3.0 kJ mol^−1^ and the respective rate constant of 10^−17^ s^−1^ at 25 °C. In the assumed model, two mechanisms of disulfide bond impact on the stability of DraE-sc are possible. The first, based on kinetic stabilization, corresponds to a higher activation free energy barrier for both folding and unfolding that results from the increase in free energy of the transition state caused by the presence of the A–B type disulfide bond. The second, based on thermodynamic stabilization, is connected with lowering of the free energy of the folded state with respect to the unfolded state, which results in a higher unfolding free energy. In this case, the disulfide bond stabilizes solely the final folded state of DraE-sc, *e.g.* by cooperative reinforcement of the β-sandwich hydrogen bonds, with negligible effect on the transition state energy.

Our analysis of the folding kinetics of DraE-sc and DraE-sc-ΔSS indicates that the presence of the disulfide bond has no effect on the energy of the transition state, thus ruling out the mechanism of kinetic stabilization. This result also suggests that the disulfide bond–induced stabilization must be almost entirely thermodynamic in nature. Indeed, quantitative results unambiguously show that the difference between the unfolding free energy of DraE-sc (Δ*G*_N→U_ = 83.5 ± 3 kJ mol^−1^) and DraE-sc-ΔSS (Δ*G*_N→U_ = 30 ± 5 kJ mol^−1^) at 25 °C is large (ΔΔ*G*_N→U_ ≅ 53.5 ± 9 kJ mol^−1^). Consequently, the kinetic stability of DraE-sc that is strictly dependent on the height of free energy barrier of unfolding, Δ*G*_N→_**_‡_**, can be entirely attributed to the disulfide bond–induced stabilization of the native state (Fig. 9). This conclusion is further confirmed by a large increase in the unfolding rate constant associated with the deletion of the disulfide bond. The unfolding rate constant of DraE-sc-ΔSS at 25 °C, *k*_unfold_ = 10^−7^–10^−8^ s^−1^, calculated as the ratio between the folding rate constant *k*_fold_ and the folding equilibrium constant *K* (10), was 9–10 orders of magnitude higher than the respective rate constant for DraE-sc. The hypothesis that the stabilizing effect of the disulfide bond is exclusively related to the stabilization of the native state was further verified by presented DraE-sc reoxidation experiments (Figs. 3 and 4). This means that the observed kinetic stability of DraE-sc, exemplified by its extremely low unfolding rate constant (10^−17^ s^−1^), simply results from its thermodynamic stability (Δ*G*_N→U_).

Structural insight into the role of the disulfide bond on DraE stability comes from the molecular dynamics simulations of thermal and mechanical unfolding of DraE-sc and DraE-sc-ΔSS proteins. Although cooperative, the unfolding process of DraE-sc-ΔSS may be divided into two steps: first, related to the dissociation of strands A and G, and second, involving denaturation of the remaining protein core (Fig. 5). Assuming that the final (non-rate-limiting) stage of folding is reversible, the initial events of unfolding should correspond to the late stages of folding. This suggests that A and G strands are the accessory strands that dock to the core-forming strands at the very end of the folding process and can stabilize the native state without affecting the stability of the transition state.

Notably, in MD simulations the disulfide bond can be seen to stabilize the structure of the native state in a cooperative manner, *i.e.* reduction of local fluctuations of strand A by covalent joining to the core strand B translates to higher global kinetic stability of the protein fold. This also suggests that the localization of the disulfide bond in the structure of DraE is crucial to its functioning under physiological conditions, *i.e.* under shear stress exerted on adhered bacteria in urine flow (Fig. 6). Attachment of the N-terminal end of the A strand to the core-forming B strand stabilizes the critical point of the subunit where mechanical force is transmitted from one subunit to the other through the donor Nte-A strand connection. The use of this A–B type disulfide bond as adaptation to functioning under shear stress is common to many bacterial adhesive structures, as is reflected in the wide prevalence of the DraE-type disulfide bond in the family of protein subunits of chaperone-usher type fimbriae (supplemental Fig. S2 and Table S1).

The indispensability of the stabilizing function of the disulfide bond was emphasized by the discovery of a specific quality control mechanism that protects fimbrial structures from incorporation of disulfide bond–deficient subunits of type 1 pili. Indeed, the FGS-type FimC chaperone binds the FimA subunit and catalyzes its folding only if the polypeptide contains a disulfide bond (15). Here, our direct *in vivo* experiments confirm the presence of a similar control mechanism in Dr fimbriae biogenesis. This is the first example of quality control of disulfide bond formation in a chaperone belonging to the FGL subfamily. It is an important result because the DSC reaction, key in fimbrial biogenesis, differs largely between FGL and FGS chaperones.

The presented mode of folding/unfolding of DraE-sc, as well as the effect of the disulfide bond, are in line with the general mode of folding described for the third fibronectin type III domain from human tenascin (TNfn3), the model protein with the Ig-like fold (20, 25). Similarly to the DraE-sc protein, mutations of residues in strand A of TNfn3 resulted in a pronounced change in the unfolding free energy, ΔΔ*G*_N→U_, and a smaller change of the activation free energy, ΔΔ*G*_U→‡_. In turn, the role of the self-complemented G strand in the folding of fimbrial subunits was analyzed experimentally for the FimA rod subunit of type 1 pili (12). The wild-type FimA protein (without the G strand) folds with the same rate constant as the recombinant self-complemented FimA-sc, indicating that G strand is not important for the formation of the rate-limiting transition state. Inversely, the unfolding rate constant of wild-type FimA is 13 orders of magnitude lower than in case of FimA-sc, indicating the high relevance of the G strand for the stability of the native state. In this regard, strand G of FimA behaves similarly to accessory strands A and G of DraE and TNfn3, docking to the formed protein core at the last stage of folding.

In conclusion, we demonstrate that the stabilizing effect of the disulfide bond in DraE-sc subunits is strictly thermodynamic, *i.e.* results from lowering of the free energy of the folded form with respect to the denatured state. This thermodynamic origin of stabilization is reflected in a lack of effect of the Cys → Ala mutations on the activation free energy for folding and a significant effect of these mutations on the unfolding free energy, which translates to a change in unfolding rate constant of 9–10 orders of magnitude. Because the A–B type disulfide bond is well conserved among the chaperone-usher type subunits of adhesive structures in Gram-negative bacteria, we speculate that the presented mechanism of stabilization may be common to the entire family.

## Experimental procedures

### Bacterial strains and plasmids

*E. coli* BL21(DE3) (Merck) was used as the host for recombinant protein production encoded by plasmids with gene controlled by T7 promoter. *E. coli* AAEC191A was used for expression of proteins involved in the assembly of Dr fimbriae. This strain does not produce type 1 pili by itself (26). All *E. coli* strains used in this work and encoded by these plasmids are summarized in supplemental Table S2.

The pET30b-sygDraBE plasmid, described previously, encodes the wild *draB* and *draE* genes in a bicistronic system (8). pET30b-sygDraBE-ΔSS, constructed in this work, derives from pET30b-sygDraBE and has cysteine codons exchanged to alanine in the *draE* gene using the PCR-borne site-directed mutagenesis kit (Stratagene). The *E. coli* BL21(DE3)/pET30b-sygDraBE-ΔSS strain under control of IPTG-inducible T7 promoter produced wild DraB chaperone and DraE fimbrial subunit lacking the disulfide bond, both of which were transported to the periplasm. The bacterial cultivation and protein expression parameters were identical as described previously for the *E. coli* BL21(DE3)/pET30b-sygDraBE strain (8).

The pCC90 plasmid encodes the dra operon with its promoter region and regulatory genes upstream of a draB gene deleted. The expression of *dra* operon is controlled by the vector promoter and results in production of surface localized wild Dr fimbriae (27). The pCC90D54stop is a derivative of pCC90 with codon D54 mutated to codon stop in the *draE* gene. Strains encoding this plasmid do not produce DraE protein (27). The pCC90DraE-ΔSS plasmid constructed in this work is a derivative of pCC90 with cysteine codons exchanged to alanine within the *draE* gene. Production of potential Dr fimbriae composed from DraE-ΔSS subunits by *E. coli* AAEC191A/pCC90DraE-ΔSS was identical as described previously (28).

pGFP stands for the commercial pSF-OXB20-daGFP (Oxford Genetics) plasmid that encodes GFP under the control of a strong constitutive promoter, OXB20. The plasmid has its origin derived from pBR322 and encodes resistance to kanamycin. The plasmid was used to detect bacterial cells *E. coli* AAEC191A/pCC90/pGFP, AAEC191A/pCC90DraE-ΔSS/pGFP, and AAEC191A/pCC90D54stop/pGFP by fluorescence microscopy.

The construction of pET30-DraE-sc and pET30-DraE-sc-ΔSS vectors that encode DraE-sc and DraE-sc-ΔSS proteins, respectively, was described in previous works (9, 10). The recombinant proteins are composed of the following segments (ordered from the N to C termini): the N-terminal signal peptide for periplasmic localization, the His tag composed of six consecutive histidine residues, and the DraE β-sandwich lacking the Nte donor strand (GFTPSGTTGTTKLTVT) and complemented at C terminus by the same Nte donor strand joined with the DNKQ linker peptide (supplemental Fig. S1).

### Protein expression and purification

The DraE-sc and DraE-sc-ΔSS proteins were produced in *E. coli* BL21(DE3)/pET30-DraE-sc and BL21(DE3)/pET30-DraE-sc-ΔSS strains identically as described earlier (9, 10). In brief, 25 ml of an overnight culture was inoculated in 0.5 liter of LB medium complemented with kanamycin at 20 μg ml^−1^; the culture was grown with agitation at 37 °C to *A*_600_ = 0.3, induced by adding IPTG to final concentration of 0.5 mm, and grown for an additional 2 h. The periplasmic fraction containing the mature DraE-sc or DraE-sc-ΔSS protein was extracted from the harvested cells. Proteins were purified by metal-affinity chromatography and size-exclusion chromatography, as described for other self-complemented periplasmic DraE constructs (9, 10).

### Protein concentrations

The protein sequences and molecular weights of all DraE variants used in this work are summarized in supplemental Fig. S1. Concentrations of recombinant proteins were measured by absorbance at 280 nm using molar extinction coefficients calculated based of amino acids sequences. Molar extinction coefficients of 24,075 m^−1^ cm^−1^ for DraE-sc and 23,950 m^−1^ cm^−1^ for DraE-sc-ΔSS were used.

### Far-UV CD spectroscopy of native and GdmCl-denatured proteins

Far-UV CD spectroscopy was used to monitor spectra of native DraE-sc and DraE-sc-ΔSS proteins and for determination of minimal GdmCl required for their denaturation (supplemental Fig. S4). CD measurements were performed using a JASCO J-815 spectropolarimeter in 1-mm quartz cuvettes thermostatted by a Peltier element at 25 °C. The spectra were recorded in the 210–250 nm range with a scan speed of 50 nm min^−1^, a bandwidth of 1 nm, and a response of 0.5 s. Six spectra were averaged and corrected for the presence of buffer. The used DraE-sc and DraE-sc-ΔSS protein samples had a final concentration of 15 μm in 20 mm sodium phosphate (pH 7.5) and 100 mm NaCl, contained different concentration of GdmCl (0–6 m), and were equilibrated before measurements for 24 h at room temperature.

### Refolding kinetics

Stock solutions of DraE-sc and DraE-sc-ΔSS (80 μm in 20 mm sodium phosphate, pH 7.5, and 100 mm NaCl) were denatured by dilution with buffer containing 7 m GdmCl, 20 mm sodium phosphate (pH 7.5) and 100 mm NaCl and equilibrated for 24 h in room temperature. The denatured samples (DraE-sc and DraE-sc-ΔSS of 11 μm, 6 m GdmCl) were concentrated using Amicon Ultracel-10K centrifugal filters with volume of 15 and 0.5 ml (Merck, Millipore, Ireland) to a final concentration of DraE-sc and DraE-sc-ΔSS of 2 mm. The refolding of chemically denatured proteins were initiated by manual mixing (1:100) with 20 mm sodium phosphate (pH 7.5) and 100 mm NaCl to final concentration of DraE-sc of 18 μm, DraE-sc-ΔSS of 20 μm, and GdmCl of 60 mm. Refolding of both proteins was monitored by recording changes in CD signal at constant wavelength of 227 nm with a bandwidth of 1 nm, a data pitch of 1 s, and a response time of 1 s for 2 h at 25 °C. Measurements were performed using a JASCO J-815 spectropolarimeter in 1-mm quartz cuvettes thermostatted by a Peltier element. The refolding kinetics of both proteins followed first-order kinetics, because the data were well fitted with the exponential function ϴ_t_/ϴ_0_ = *e*^−kt^. Refolding of DraE-sc and DraE-sc-ΔSS proteins was also monitored by recording far-UV CD spectra in rage from 250 to 203 nm with a scan speed of 50 nm min^−1^, a bandwidth of 1 nm, and a response time of 0.5 s for 1 h at 25 °C.

### Oxidation of cysteine residues to disulfide bond in the DraE-sc

Recombinant DraE-sc at concentration of 20 μm was denatured in 20 mm Tris-HCl (pH 8.0), 0.2 m NaCl, 50 mm DTT, and 6 m GdmCl for 12 h in room temperature. Then the sample was concentrated to final DraE-sc concentration of ∼10 mm using Amicon Ultracel-10K centrifugal filters with volume of 15 and 0.5 ml (Merck, Millipore). The refolding reaction of chemically denatured and reduced DraE-sc protein was initiated by its 100-fold dilution with refolding buffer: 20 mm Tris-HCl (pH 8.0), 0.2 m NaCl, and 10 mm DTT, to a final concentration of DraE-sc of 90 μm, GdmCl of 60 mm, and DTT of 15 mm. The refolding was conducted for 6 h in room temperature. Then the refolding reaction was divided into three equal samples labeled as 1, 2, and 3. DTT was added to sample 3 to a final concentration of 100 mm; CuCl_2_ was added to sample 2 to a final concentration of 2 mm; no additives were added to sample 1. The three samples were incubated for 7 days at room temperature. After every 24 h, each sample was analyzed by SDS-PAGE retardation assay to detect air-oxidized DraE-sc (*i.e.* containing the disulfide bond). The gels were stained with Coomassie Blue R-250, digitalized to TIF files using Epson Perfection V750 PRO scanner, and then quantified with SCION IMAGE software.

### Microcalorimetry

Calorimetric experiments were performed on the CSC 6300 Nano-DSC III differential scanning microcalorimeter (Calorimetry Sciences Corp., Lindon, UT) with a capillary cell volume of 0.299 ml in the temperature range from 5 to 95 °C. The experimental data were recorded using DSCRun (Calorimetry Sciences Corp.). The concentration of refolded reoxidized (containing disulfide bond) and refolded reduced (containing –SH groups) DraE-sc (molecular mass of 16.3 kDa) was 1.0 mg ml^−1^ in each experiment. The analysis was performed with a scanning rate of 0.5, 1.0. 1.5, and 2.0 °C min^−1^. Samples preparation, measurements, and data analysis were performed as described previously for the self-complemented DraE-sc protein (9, 10).

### Bacterial adherence to urinary bladder cells and immunofluorescence microscopy

The T 24 (ATCC HTB-4) urinary bladder cancer cells were purchased from ATCC-LGS. The cells were cultivated in McCoy's 5a growth medium (ATCC-LGS) supplemented with FBS (ATCC-LGS) to a final concentration of 10% and penicillin-streptomycin solution (Sigma) according to the producer culturing method. For the adherence assay, the bladder cells were growth to confluent monolayer in 35-mm polystyrene dishes (Corning). The overnight LB cultures of *E. coli* AAEC191A/pCC90/pGFP, AAEC191A/pCC90DraE-ΔSS/pGFP, and AAEC191A/pCC90D54stop/pGFP were centrifuged at 2,000 × *g* and suspended in McCoy's 5a medium supplemented with 0.5% BSA to final optical density *A*_600_ of 0.1. To check whether bacterial adherence is sensitive to the presence of chloramphenicol, the bacterial suspensions were preincubated in 300 μm chloramphenicol before experiment for 10 min at room temperature. Bacterial adherence to bladder cells was performed in two modes. In stationary mode, 1 ml of bacterial suspension was added to dishes with a cell line washed with fresh medium. Then the dishes were incubated in cell culture incubator for 20 min. After this time, non-bound bacteria were washed away with medium. In dynamic mode, to the top of the dish with the cell culture, washed with fresh medium, a flow chamber was assembled with a 2-cm-long, 2.5-mm-wide, and 0.25-mm-thick gasket (Glycotech). The bacterial suspension was passed through the flow chamber to generate shear stress of 0.1 pN μm^−2^ for 20 min at room temperature, using a syringe pump (Harvard Apparatus). Then the pump was switched to culture medium for 2 min to wash out non-bound bacteria. Adherence of GFP-producing bacteria to bladder cells was monitored by recording green fluorescence using Olympus IX73 P2F inverted fluorescence microscope under UCPLFLN PH 20×/0.70 objective and equipped with Hamamatsu Orca-flesh2.8 digital camera. The photos were collected and analyzed using Olympus cellSens Dimension 1.15 and Imaris ×64 (Bitplane) software, respectively. For each dish, 15 photos with fields of view of 350 × 262 μm were recorded. All experiments were repeated at last three times in triplicate. The number of adherent bacteria was expressed as means ± S.E. of the means, and statistical significance was assessed by Student's *t* test (*p* < 0.05 was considered to be significant).

The presence of Dr fimbria on the surface of *E. coli* AAEC191A/pCC90 and AAEC191A/pCC90DraE-ΔSS strains and control strain AAEC191A/pCC90D54stop was monitored using immunofluorescence microscopy. The bacterial cultures grown on LB at 37 °C for 24 h were centrifuged, washed gently, and suspended in PBS. Bacterial suspensions (10^5^–10^6^ cells ml^−1^) were incubated with rabbit anti-DraE primary antibodies (Immunolab, Gdynia, Poland) diluted 1:500 at room temperature for 1 h. The reaction mixtures were then washed three times with PBS containing 10% (v/v) glycerol and incubated with goat anti-rabbit IgG-TRITC conjugate of secondary antibodies (Sigma-Aldrich) diluted 1:5000 at room temperature for 1 h. The reaction mixtures were then washed again three times with PBS containing 10% (v/v) glycerol. Bacterial suspensions were loaded on glass slides and observed with microscope identically as for adherence assays.

### SDS-PAGE retardation assay, Western blot analysis, periplasmic fractions preparation, analytical gel filtration, and other techniques

SDS-PAGE retardation assay was carried out by using samples mixed with Laemmli buffer that were incubated at 25 °C or heated at denoted temperature for 10 min. Electrophoresis was performed in 15% polyacrylamide gels in running buffer (0.1% SDS and Tris-glycine, pH 8.3). After electrophoresis, gels were stained using Coomassie Blue or used in Western blotting.

Immunoblot detection of DraE protein was performed with polyclonal rabbit monospecific anti-DraE antibodies (Immunolab) diluted 1:2000 and secondary goat anti-rabbit antibodies labeled with horseradish peroxidase (Sigma-Aldrich) diluted 1:5000 and diaminobenzidine as a reaction substrate. Periplasmic fractions of *E. coli* BL21(DE3)/pET30b-sygDraBE and BL21(DE3)/pET30b-sygDraBE-ΔSS strains were extracted by the osmotic shock procedure (29).

Analytical size-exclusion chromatography of reduced and oxidized forms of DraE-sc proteins was performed using Superdex 75 10/300GL column (GE Healthcare, UK). The column was calibrated using LMW gel filtration calibration kit (GE Healthcare). Column void volume was determined with blue dextran 2000 (GE Healthcare). Detection of disulfide bonds was performed using a non-direct method with Ellman's reagent, 5,5′-dithiobis(2-nitrobenzoic acid) (Sigma), as described previously (8).

### Molecular dynamics simulations

All fully atomistic MD simulations were based on the X-ray structure of the self-complemented AfaE subunit (98% identity to DraE that translate into three differences in amino acid sequence) found in Protein Data Bank entry 1RXL. In the mechanical unfolding simulations, three N-terminal residues (EEC) were fused additionally to the C terminus to provide an additional handle for the pulling force. In case of thermal unfolding simulations, the C terminus was instead extended with the whole G strand (residues 128–146) to more closely emulate the experimental setup. The protein model was prepared in two versions: oxidized, with the disulfide bond connecting cysteines 3 and 35, and reduced, in which these two residues are not covalently bonded. In all simulations, the CHARMM36 force field was used as implemented in Gromacs 5.0.4 (30, 31). The constructed systems consisted of a single protein molecule embedded in a cubic periodic box with box vector length of 11.3 or 7.5 nm, solvated with 34047 or 13019 TIP3P water molecules and 0.154 m potassium chloride added to neutralize the net charge in case of thermal and mechanical unfolding simulations, respectively. Five thermal denaturation simulations were carried out for each version of the protein (oxidized and reduced), in which the system was subject to fast initial annealing (from 300 to 450 K) during the first 50 ns and subsequently warmed up more slowly (from 450 to 480 K) over the remaining 250 ns. This choice was motivated by preliminary simulations at 440 K, in which the oxidized form of DraE-sc resisted thermal unfolding for over 4 μs. For the steered MD (mechanical unfolding) simulations, 10 independent 500-ns simulations were ran for each version of the protein, with the pulling force applied between the centers of mass of the C- and N-terminal three-residue EEC motifs using Plumed (32). The force constant was set to 5000 kJ mol^−1^ nm^−1^, and the pulling speed was adjusted so that over the 500-ns simulation the end-to-end distance between the two termini increased by 3.5 nm.

## Author contributions

J. P. performed and analyzed experiment of reoxidation of DraE-sc protein shown in Fig. 6. B. Z.-P. co-designed the study, constructed recombinant strains, and performed the experiment shown in Figs. 2 and 3. P. B. analyzed the far-UV CD and DSC experimental data shown in Figs. 5 and 7 and wrote the paper. J. C. and M. W. designed, performed, and analyzed the MD experimental data shown in Figs. 8 and 9 and wrote the paper. M. O. produced recombinant proteins. M. W. performed and analyzed analytical size-exclusion chromatography. B. N. analyzed the results and approved the final version of the manuscript. D. A.-N. performed far-UV CD experiments. R. P. designed the study, wrote the paper, and performed DSC, fluorescence microscopy, and WB experiments. All authors reviewed the results and approved the final version of the manuscript.

## Supplementary Material

Supplemental Data

## Footnotes

2 The abbreviations used are:

DSC: donor strand complementation mechanism
DSE: donor strand exchange
FGL: long F1-G1 loop
FGS: short F1-G1 loop
GdmCl: guanidinium hydrochloride
MD: molecular dynamics
Nte: N-terminal extension
RMSD: root mean square deviation
IPTG: isopropyl β-d-thiogalactopyranoside.
