# Supplementary material for: Role of the disulfide bond in stabilizing and folding of the fimbrial protein DraE from uropathogenic Escherichia coli
Source: J Biol Chem. 2017 Jul 24;292(39):16136–49. doi: 10.1074/jbc.M117.785477 (PMC5625045; doi:10.1074/jbc.M117.785477)
Supplement: Supplemental Data [file supp_292_39_16136__index.html]

Role of the disulfide bond in stabilizing and folding of the fimbrial protein DraE from uropathogenic Escherichia coli — Role of the disulfide bond in stabilizing and folding of the fimbrial protein DraE from uropathogenic Escherichia coli — Folding and stability of DraE adhesin — Supplemental Data 

# Role of the disulfide bond in stabilizing and folding of the fimbrial protein DraE from uropathogenic *Escherichia coli*

## Supplemental Data

- Supplemental data (.pdf, 829 KB) - Supplemental data including: text, tables, figures and references.
- movie 1 (.avi, 12.4 MB) - Non
- movie 2 (.avi, 12.8 MB) - Non
