# Supplementary material for: Role of the disulfide bond in stabilizing and folding of the fimbrial protein DraE from uropathogenic Escherichia coli
Source: J Biol Chem. 2017 Jul 24;292(39):16136–49. doi: 10.1074/jbc.M117.785477 (PMC5625045; doi:10.1074/jbc.M117.785477)
Supplement: Supplemental Data [file 10.1074_M117.785477_jbc.M117.785477-1.pdf]

Supplemental Data for:

Role of the disulfide bond in stabilizing and folding of the fimbrial protein DraE from uropathogenic *Escherichia coli*

Justyna Pilipczuk[1], Beata Zalewska-Piątek[1], Piotr Bruździak[2], Jacek Czub[2], Miłosz Wieczór[2], Marcin Olszewski[1], Marta Wanarska[1], Bogdan Nowicki[3], Danuta Augustin-Nowacka[4] and Rafał Piątek[1*]

[1]Department of Molecular Biotechnology and Microbiology, Gdańsk University of Technology, ul. Narutowicza 11/12, 80-233 Gdańsk, Poland

[2]Department of Physical Chemistry, Gdańsk University of Technology, ul. Narutowicza 11/12, 80-233 Gdańsk, Poland

[3] Nowicki Institute for Women's Health Research (Now I for HeR), 114 Governors Way, Brentwood, TN 37027, USA

[4]Faculty of Chemistry, University of Gdańsk, ul. Wita Stwosza 63, 80-308 Gdańsk, Poland

Supplemental Data:

Supplemental text

Two supplemental tables with legend

Four supplemental figures with legends

Supplemental references

Two supplemental movies files

Supplemental text

Statistics of cysteine positions in fimbrial subunit sequences

Protein sequences belonging to the major fimbrial subunit families (as described in Nuccio and Bäumler, 2007) were batch downloaded from the PFAM and COG databases and duplicates (defined with 100% sequence similarity) were removed (1). To exclude proteins that do not share a common fold with DraE, a sequence length criterion (121-171 amino acids, ± 25 with respect to length of DraE-sc) was used based on the sequence length distribution. For each thus selected protein, normalized cysteine positions were calculated as the consecutive number of the cysteine in the sequence divided by the sequence length, so that the number corresponds to an interval between 0 and 1. Histograms were then simply obtained by binning the calculated normalized positions. Table S1 provides an estimate of the prevalence of A-B DraE-like and B-F DraD-like disulfides in individual families; here, a simple criterion was used to discern A-B type disulfide bonds (two cysteines with normalized position < 0.5) from B-F disulfide bonds (one cysteine with normalized position < 0.5 and one with > 0.5).

Φ-value Analysis of Cys→Ala Mutation on the Energy of DraE-sc Folding Transition State

The Φ-value is defined by the following equation:

$$\Phi_F = \frac{\Delta\Delta G_{U\to ‡}}{\Delta\Delta G_{U\to N}}$$

, where $\Delta\Delta G_{U\to ‡}$ and $\Delta\Delta G_{U\to N}$ are the disulfide bond-induced change in the free energy difference between U and ‡ states for folding and the change in the free energy difference between U and N states, respectively (Fig. 9). A Φ_F value of 1 means that the free energy of the transition state is equally perturbed by the mutation as the free energy of the folded state. On the contrary, a Φ_F value of 0 indicates that in the transition state, the residue of interest has not yet assumed a native-like structure and is located in an unfolded region (2). The Φ_F value

for the two Cys → Ala mutations in DraE-sc is 0.036 ± 0.015, which confirms the marginal effect of the mutation on the free energy and structure of the transition state. It is worth noting that the Φ_F parameter is not dependent on the influence of the analyzed mutation on the energy of the unfolded state.

Supplemental tables

Family ID	# of proteins	# length in range 121-171	# of putative A-B	% members with A-B	# of putative B-F	% members with B-F
**PF06551**	1126	1	1	100.00	0	0.00
**PF05775**	124	0	0	0.00	0	0.00
**PF04619**	85	80	79	98.75	1	1.25
**PF02432**	467	26	0	0.00	0	0.00
**PF06443**	53	28	0	0.00	0	0.00
**PF00419**	18453	16140	15206	94.21	7	0.04
**COG3539**	439	23	12	52.17	2	8.70
**PF07434**	425	28	3	10.71	24	85.71
**COG5430**	237	60	35	58.33	0	0.00
**PF04449**	666	514	1	0.19	0	0.00

Table S1. Statistics of protein sequences used in sequence analysis. Individual columns contain (1) the ID of the protein family in the PFAM (Protein FAMily) and COG (Clusters of Orthologous Groups) databases; (2) total number of protein sequences in the respective family; (3) number of protein sequences that satisfy the sequence length criterion; (4), (5) estimated number and percentage of proteins from (3) that contain the A-B disulfide bond; (6), (7) estimated number and percentage of proteins from (3) that contain the B-F disulfide bond.

E. coli bacterial strain	Encoded plasmid(s)	Antibiotic resistance	Expressed recombinant proteins *	Expression inductor	Ref.
BL21(DE3)/ pET30b-sygDraBE	pET30b-sygDraBE	Kanamycin	DraB, DraE	IPTG	(3)
BL21(DE3)/ pET30b-sygDraBE-ΔSS	pET30b-sygDraBE-ΔSS	Kanamycin	DraB, DraE-ΔSS	IPTG	#
AAEC191A/ pCC90	pCC90	Ampicillin	DraB, DraC, DraD, DraE	Constitutive	(4)
AAEC191A/ pCC90DraE-ΔSS	pCC90DraE-ΔSS	Ampicillin	DraB, DraC, DraD, DraE-ΔSS	Constitutive	#
AAEC191A/ pCC90D54stop	pCC90D54stop	Ampicillin	DraB, DraC, DraD	Constitutive	(4)
AAEC191A/ pCC90/pGFP	pCC90<br>pSF-OXB20-daGFP **	Ampicillin<br>Kanamycin	DraB, DraC, DraD, DraE<br>GFP	Constitutive<br>Constitutive	(4)
AAEC191A/ pCC90DraE-ΔSS/pGFP	pCC90DraE-ΔSS<br>pSF-OXB20-daGFP	Ampicillin<br>Kanamycin	DraB, DraC, DraD, DraE-ΔSS<br>GFP	Constitutive<br>Constitutive	#
AAEC191A/ pCC90D54stop/ pGFP	pCC90D54stop<br>pSF-OXB20-daGFP	Ampicillin<br>Kanamycin	DraB, DraC, DraD<br>GFP	Constitutive<br>Constitutive	(4)

Table S2. Characterization of used *E. coli* bacterial strains. (*) - denoted are only proteins important for experiments, other vector encoded proteins are omitted; (**) - in the publication text the pSF-OXB20-daGFP (Oxford Genetics Ltd, UK) was signed as pGFP; (#) – plasmids constructed in this work.

Supplemental Figures

DraE, molecular weight of mature protein: **14.9 kDa**

MKKLAIMAAASMVFAVSSAHAGFTPSGTTGTTKLTVTEECQVRVGDLTVAKTRGQLTDAAPIGPVTVQALGCDARQVALKADTDNFEQGKFFLISDNNRDKLYVNIRPTDNSAWTTDNGVFYKNDVGSWGGIIGIYVDGQQTNTPPGNYTLTLTGGYWAK

DraE-ΔSS, molecular weight of mature protein: **14.9 kDa**

MKKLAIMAAASMVFAVSSAHAGFTPSGTTGTTKLTVTEEAQVRVGDLTVAKTRGQLTDAAPIGPVTVQALGADARQVALKADTDNFEQGKFFLISDNNRDKLYVNIRPTDNSAWTTDNGVFYKNDVGSWGGIIGIYVDGQQTNTPPGNYTLTLTGGYWAK

DraE-sc, molecular weight of mature protein: **16.3 kDa**

MKKLAIMAAASMVFAVSSAHAHHHHHHAEECQVRVGDLTVAKTRGQLTDAAPIGPVTVQALGCDARQVALKADTDNFEQGKFFLISDNNRDKLYVNIRPTDNSAWTTDNGVFYKNDVGSWGGIIGIYVDGQQTNTPPGNYTLTLTGGYWAK**DNKQGFTPSGTTGTTKLTVT**

DraE-sc-ΔSS, molecular weight of mature protein: **16.3 kDa**

MKKLAIMAAASMVFAVSSAHAHHHHHHAEEAQVRVGDLTVAKTRGQLTDAAPIGPVTVQALGADARQVALKADTDNFEQGKFFLISDNNRDKLYVNIRPTDNSAWTTDNGVFYKNDVGSWGGIIGIYVDGQQTNTPPGNYTLTLTGGYWAK**DNKQGFTPSGTTGTTKLTVT**

Figure S1. Protein sequences of used DraE variants. Used fonts signs: black underlined – N-terminal signal sequence governing protein transport to periplasm, signal peptide is not present in mature protein; red – adhesin N-terminal extension (Nte) required for donor strand exchange (DSE) reaction; green – His-Tag fusion peptide; violet – peptide linker used to join fusion G strand; blue fonts – C-terminal fusion G strand that complemented protein Ig-like fold; black on yellow background – cysteine residues forming A-B type disulfide bond that in DraE-ΔSS DraE-sc-ΔSS proteins are changed to alanine.

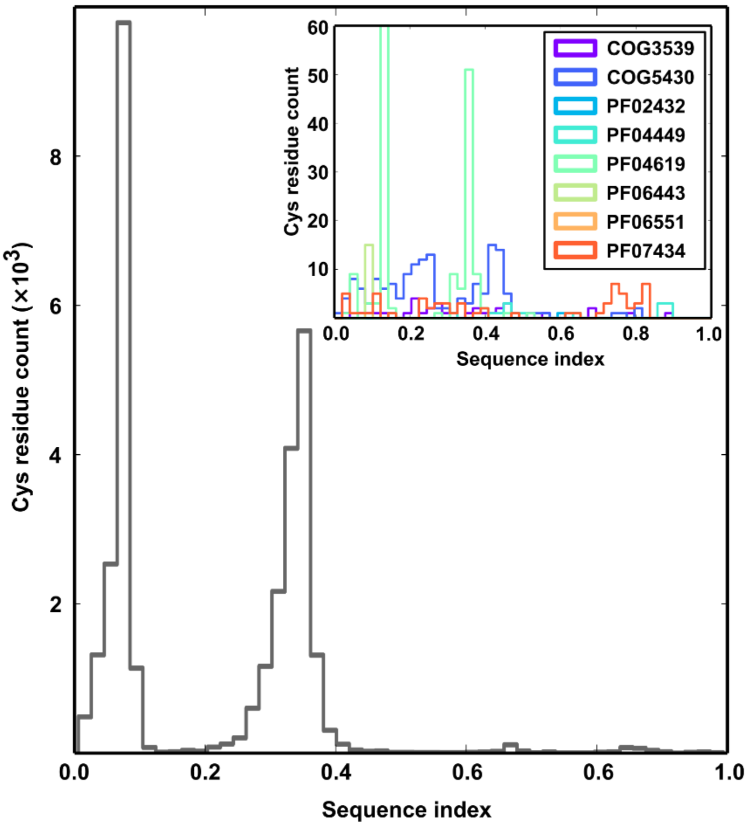

Figure S2. Histogram of cysteine positions in sequences of fimbrial subunits classified into different COG and PFAM families (selected according to Nuccio and Bäumler, 2007, (1)). The main histogram (gray) corresponds to main fimbrial family PFAM00419. Sequence index is defined as the normalized position in a sequence that assumes values between 0 (N-terminal end) and 1 (C-terminal end); see Supplemental text above for further details, and Table S2 for an estimate of the prevalence of different disulfide bond variants in individual families. Typically, the A-B disulfide bond is formed by cysteines found at sequence indices of ca. 0.075 and 0.35.

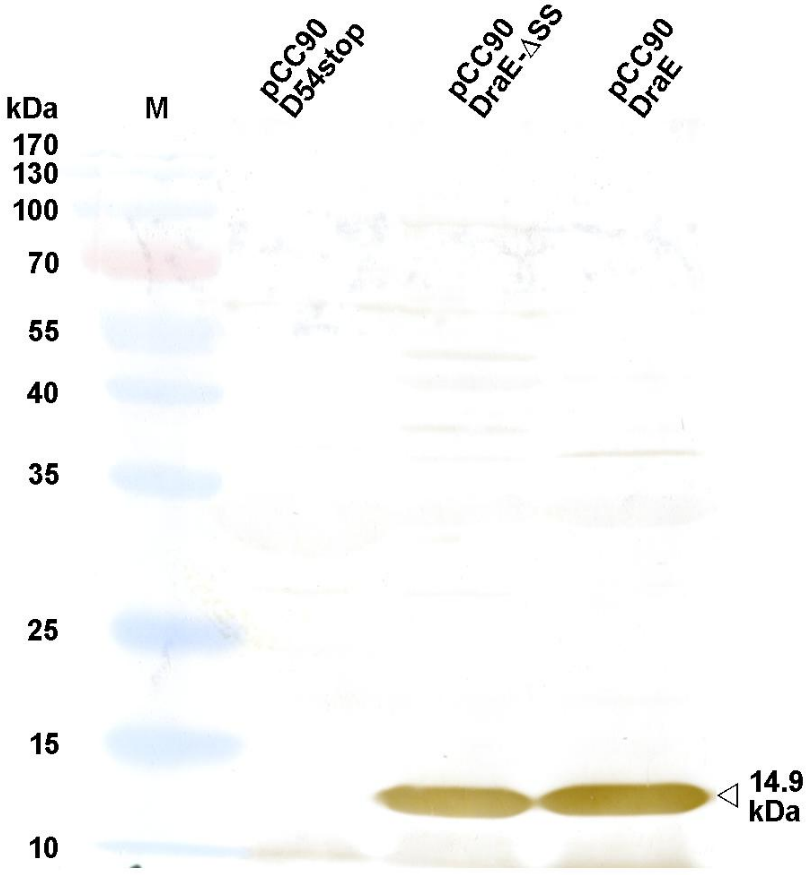

Figure S3. Western blot analysis of periplasmic fractions with anti-DraE antibodies. Fractions were isolated from *E. coli* AAEC191A/pCC90D54stop, AAEC191A/pCC90DraE-ΔSS and AAEC191A/pCC90 strains. Samples were incubated with Laemmli buffer at 100 °C for 10 minutes, followed by electrophoresis (SDS—15% polyacrylamide gel). The triangle denotes bands corresponding to the mature DraE-ΔSS and DraE proteins. Lane M contained a PageRuler Prestained Protein Ladder (Fermentas) which included 10, 15, 25, 35, 40, 55, 70, 100, 130 and 170 kDa compounds.

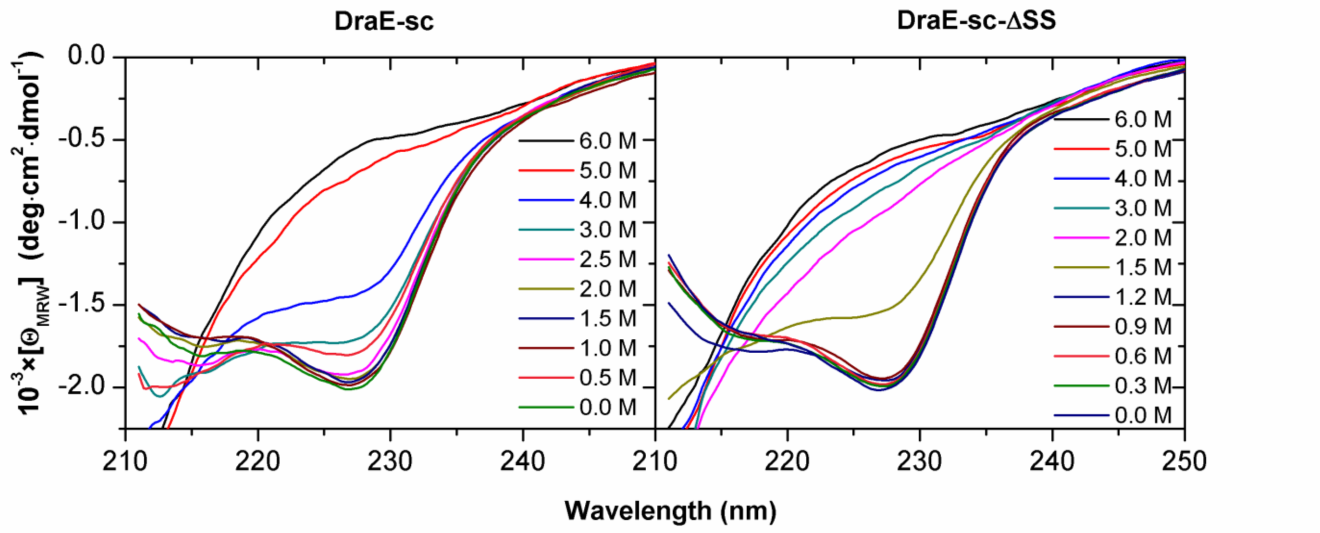

Figure S4. far-UV CD spectra of DraE-sc and DraE-sc-ΔSS proteins equilibrated in 20 mM sodium phosphate (pH 7.5) and 100 mM NaCl, contained different concentration of GdmCl (0 – 6 M). Molarities of GdmCl are given in legends. The spectra of DraE-sc-ΔSS protein in buffers containing more than 1.5-2 M GdmCl are typical for a fully denatured protein. At the same conditions, spectra of DraE-sc are indicative of the retention of native structure at up to 3 M GdmCl, with full denaturation above 5 M GdmCl.

Supplemental References

1. Nuccio, S. P., and Bäumler, A. J. (2007) Evolution of the chaperone/usher assembly pathway: fimbrial classification goes Greek. *Microbiol. Mol. Biol. Rev.* **71**, 551-575
2. Hamill, S., Steward, A., and Clarke, J. (2000) The folding of an immunoglobulin-like Greek key protein is defined by a common-core nucleus and regions constrained by topology. *J Mol Biol.* **297**, 165-178
3. Piątek, R., Zalewska, B., Kolaj, O., M., F., Nowicki, B., and Kur, J. (2005) Molecular aspects of biogenesis of Escherichia coli Dr Fimbriae: characterization of DraB-DraE complexes. *Infect. Immun.* **73**, 135-145
4. Carnoy, C., and Moseley, S. (1997) Mutational analysis of receptor binding mediated by the Dr family of Escherichia coli adhesins. *Mol Microbiol.* **23**, 365-379
